# Polymorphism and structure of style–specific arabinogalactan proteins as determinants of pollen tube growth in *Nicotiana*

**DOI:** 10.1186/s12862-017-1011-2

**Published:** 2017-08-10

**Authors:** Andrzej K. Noyszewski, Yi-Cheng Liu, Koichiro Tamura, Alan G. Smith

**Affiliations:** 1 Department of Horticultural Science, University of Minnesota, 356 Alderman Hall 1970 Folwell Av., St. Paul, MN 55108 USA; 2 Present Address: Arog Pharmaceuticals, Inc, 5420 LBJ Freeway, Suite 410, Dallas, TX 75240 USA; 3 Department of Biological Sciences, Tokyo Metropolitan University, 1-1 Minami-ohsawa, Hachioji, Tokyo, 192–0397 Japan

**Keywords:** Pollen-style interactions, Intrinsically disordered proteins, Ole e 1-like domain, Positive selection

## Abstract

**Background:**

Pollen tube growth and fertilization are key processes in angiosperm sexual reproduction. The transmitting tract (TT) of *Nicotiana tabacum* controls pollen tube growth in part by secreting pistil extensin-like protein III (PELPIII), transmitting-tract-specific (TTS) protein and 120 kDa glycoprotein (120 K) into the stylar extracellular matrix. The three arabinogalactan proteins (AGP) are referred to as stylar AGPs and are the focus of this research. The transmitting tract regulates pollen tube growth, promoting fertilization or rejecting pollen tubes.

**Results:**

The N-terminal domain (NTD) of the stylar AGPs is proline rich and polymorphic among *Nicotiana* spp. The NTD was predicted to be mainly an intrinsically disordered region (IDR), making it a candidate for protein-protein interactions. The NTD is also the location for the majority of the predicted *O-*glycosylation sites that were variable among *Nicotiana* spp. The C-terminal domain (CTD) contains an Ole e 1-like domain, that was predicted to form beta-sheets that are similar in position and length among *Nicotiana* spp. and among stylar AGPs. The TTS protein had the greatest amino acid and predicted *O*-glycosylation conservation among *Nicotiana* spp. relative to the PELPIII and 120 K. The PELPIII, TTS and 120 K genes undergo negative selection, with d_n_/d_s_ ratios of 0.59, 0.29 and 0.38 respectively. The d_n_/d_s_ ratio for individual species ranged from 0.4 to 0.9 and from 0.1 to 0.8, for PELPIII and TTS genes, respectively. These data indicate that PELPIII and TTS genes are under different selective pressures. A newly discovered AGP gene, *Nicotiana tabacum* Proline Rich Protein (NtPRP), was found with a similar intron-exon configuration and protein structure resembling other stylar AGPs, particularly TTS.

**Conclusions:**

Further studies of the NtPRP gene are necessary to elucidate its biological role. Due to its high similarity to the TTS gene, NtPRP may be involved in pollen tube guidance and growth. In contrast to TTS, both PELPIII and 120 K genes are more diverse indicating a possible role in speciation or mating preference of *Nicotiana* spp. We hypothesize that the stylar AGPs and NtPRP share a common origin from a single gene that duplicated and diversified into four distinct genes involved in pollen-style interactions.

**Electronic supplementary material:**

The online version of this article (doi:10.1186/s12862-017-1011-2) contains supplementary material, which is available to authorized users.

## Background

Pollen-pistil interactions are dynamic, complex and spatially differentiated. The pollen tube delivers the male gamete to the female gametophyte, beginning with pollen grain hydration and germination on the stigma. The transmitting tract regulates pollen tube growth, promoting fertilization (plant compatibility) or rejecting pollen tubes (incompatibility). The stigma, style and TT have a role in genetic isolation of plant populations and consequently species evolution [65]. Plants evolved multiple prezygotic mechanisms to control fertilization. Self-incompatibility (SI) is a barrier that helps maintain species genetic variation [3] and interspecific incompatibility (II) prevents gene flow among species, preserving species genetic integrity.

The highly differentiated TT evolved with enclosed ovules of angiosperms and is a pathway for pollen tube growth from the stigma to the ovules [9, 42]. Pollen tubes grow rapidly [53] and the fastest growing pollen tubes reach the ovules first giving rise to progeny [4, 16], making pollen tube growth a key step where natural selection may act [43]. The initial rate of pollen tube growth through the style is slower but increases as it grows [77]. This is associated with the transition from autotrophic (nutrients obtained from the pollen grain) to heterotrophic growth (nutrients obtained from transmitting tract; [15, 37, 38, 50]). The final step of pollen tube growth is pollen tube-synergid attraction [31, 33] and finally fertilization [1, 46].

Arabinogalactan proteins are found in the plasma membrane, the cell wall, as well as the apoplastic space of the pollen tube [20, 52, 61] and are involved in many diverse processes [70]. Stylar AGPs are very abundant in the TT extracellular matrix and heterogeneous due to post-translational modifications [23, 56, 69]. The AGPs belong to a family of structurally related glycoproteins/proteoglycans, the Pro/Hyp-rich glycoproteins with attached peripheral sugars that produce large protein diversity [20]. Stylar AGPs have a hydroxyproline rich, highly *O*-glycosylated NTD and a cysteine-rich CTD [2, 86]. Three *Nicotiana* spp. AGPs, class III pistil extension-like protein (PELPIII), transmitting tissue-specific proteins (TTS) and 120 kDa protein (120 K), accumulate in the extra cellular matrix, interact with growing pollen tubes, and are developmentally regulated and involved in regulation of pollen tube growth [26, 88]. de Graaf [14] showed that the *N. tabacum* PELPIII (pMG15) CTD, in particular the cysteine pattern was highly similar to this of *N. alata* 120 K, *N. alata* PELPIII*, N. alata* GaRSGP, *Phaseolus vulgaris* PvPRP1*,* and *N. tabacum* TTS-1. It was suspected that the PELPIII gene has two exons, but the CTD of the gene was not fully described previously [14]. Current genomic resources of *N. tabacum*, both ancestral species *N. sylvestris* and *N. tomentosiformis* [73, 74] provide the possibility to fully describe intron-exon configuration of stylar AGPs.

The PELPIII protein is incorporated into the pollen tube wall of both compatible and incompatible pollen tubes [10, 13, 26]. Gardner et al., [25] produced a transmitting tract ablated line (TT-ablated) of *N. tabacum* that does not have a mature TT and has greatly reduced accumulation of the stylar AGPs. The TT-ablated line was used as a female in controlled pollinations with several species of *Nicotiana*. *Nicotiana tabacum* pollen tube growth occurred, albeit at a slightly reduced rate, suggesting the TT and AGPs are not essential for self-pollen tube growth. However, TT-ablation in *N. tabacum* did alter interspecific pollen tube growth and was essential for II with *N. obtusifolia* and *N. repanda* [76]. [18] showed that PELPIII was not essential for self *N. tabacum* pollen tube growth or seed set, but was essential for inhibition of *N. obtusifolia* and *N. repanda* pollen tube growth. Eberle et al., [17] found that *N. obtusifolia* and *N. repanda* pollen tubes grew significantly longer in *N. tabacum* styles where expression of PELPIII was suppressed*.* The TTS protein promotes self *N. tabacum* pollen tube growth in vivo and in vitro and acts as a chemical attractant for *N. tabacum* pollen tubes [7, 85]. During growth through the style, pollen tubes walls incorporate TTS and de-glycosylate it and possibly use the freed arabinogalactan as a source of energy [7]. The 120 K protein is localized to the lumen and vacuolar membranes in *N. alata* pollen tubes [27] and was shown to be required for S-specific pollen rejection [30]. Plants with no detectable PELPIII or 120 K and greatly reduced TTS all set self-seed, although pollen tube growth was reduced in plants with lower TTS accumulation [7, 17, 30]. In higher plants, the *S*-RNase is the female component of SI and the S-locus F-box is the male interactor [54]. While the 120 K is essential for *S*-specific pollen rejection, two other stylar AGPs (PELPIII and TTS) were found as *S*-RNase binding proteins [10]. Despite their abundance and regulatory functions, little is known regarding the specific mechanism of stylar AGP action in relation to pollen tube-style interactions [20, 56].

Arabinogalactan proteins undergo extensive *O-*glycosylation at hydroxyproline and serine residues [59, 80], which leads to the different molecular weights of stylar AGPs [2, 17, 30, 87]. A difference between PELPIII and TTS is the presence of repeating units of P_3–6_ in the NTD of PELPIII that are absent in TTS. Those repeats were predicted to be sites of post-translational modifications [2, 13] such as *O-* and *N-*glycosylation and may be important to AGP function in regulating pollen tube growth. However, Bosch et al., [2] found that PELPIII was not *N*-glycosylated. The level of *O*-glycosylation of TTS is higher at the top of the style than at the bottom [87]. The 120 K glycosylation patterns among closely related *Nicotiana* spp. showed differences in protein molecular weights that may result from differences in protein sequence rather than being the result of differential glycosylation [30]. de Graaf et al., [13] concluded that glycoproteins with homologous amino acid sequences may have different functions based on their distinct post-translational *O*-glycosylation patterns, which can be developmentally and spatially regulated. Algorithms for predicting plant-specific *O*-glycosylation are not fully developed; however, a consensus amino acid motif of [ASTV]-P(1,4)-X(0,10)-[ASTV]-P(1,4) was proposed by Gomord et al. [28] and can be useful in predicting *O*-glycosylation patterns of the stylar AGPs. There is evidence that *O-*glycosylation, phosphorylation and acetylation (but not *N*-glycosylation) occurs predominantly in the intrinsically disordered regions (IDRs) of plant proteins, making them hot spots for post-translational modifications [40, 41, 58]. Since *O-*glycosylation is common and associated with AGP functions [70, 71] it is important to make use of predictions for *O*-glycosylation sites to better understand AGP-protein interactions and their function in pollen tube-style interactions.

A feature of the stylar AGPs is the conserved CTD and less conserved proline-rich NTD [13, 26]. The cysteine-rich CTD shows high similarity to the conserved Ole e 1 domain [55, 68] that was first identified as the main allergen from olive pollen as well as growing pollen tubes [44, 83]. Pollen Ole e 1 was localized in extracellular space in close proximity of the pollen tube wall [12]. Muschietti et al., [55] hypothesized that Ole e 1 proteins participate in pollen tube emergence and guidance based on sequence similarities between Ole e 1 protein from olive and its homolog in tomato, the LAT52 gene. de Dios et al., [12] found a significant increase of Ole e 1 protein during and after pollen tube germination. The *N. tabacum* PELPIII, 120 K and TTS each has a conserved Ole e 1-like domain [26, 68]. Similar to the stylar AGPs, the pollen Ole e 1 protein is glycosylated, resulting in multiple glycosylation variants [12]. The petunia PhPRP1 protein has high similarity to *N. alata* NaTTS (83%) and *N. tabacum* TTS-1 (81%) and has six conserved cysteine residues of an Ole e 1-like domain, further confirming conservation of this domain outside of Oleaceae family, and its common presence in Solanaceae.

Reproductive proteins that mediate sexual reproduction by taking part in gamete recognition diverge rapidly due to adaptive evolution [49, 78]. Speciation genes prevent gene flow among populations that can result in the divergence of populations, creation of new species and prevent inbreeding depression. Signatures of natural selection [57] are identified by comparing orthologous genes and provide insights into adaptation and the processes of speciation [84]. Rapid gene evolution (gene sequence divergence) would be indicative of natural selection acting on a gene. The d_n_/d_s_ ratio (where: d_n_ = rate of nonsynonymous substitution; d_s_ = rate of synonymous substitution) is a method to quantify how amino acid changes accumulate during the course of evolution [36]. A high d_n_/d_s_ ratio (above 1) suggests that adaptive evolution has been frequent with a high rate of functional protein divergence arising from positive selection [19]. The gametophytic SI locus, the *S* locus in Solanaceae encodes a RNase and has a signature of positive selection with a d_n_/d_s_ ratio greater then 1 [64]. Interspecific incompatibility and SI in *Nicotiana* spp. act as prezygotic isolation mechanisms [51]. The stylar AGPs, particularly PELPIII and 120 K take part in II and SI, respectively [17, 30] and are excellent candidates to test whether they have undergone positive selection. In contrast, TTS is known to take part in regulation of pollen-tube growth and it could have a distinct d_n_/d_s_ ratio when compared to PELPIII and 120 K, as proteins involved in II and SI, respectively.

To better understand the role that stylar AGPs play in sexual reproduction, the regulation of pollen tube growth and the mechanisms of reproductive barriers, PELPIII (12 species) and TTS (10 species) cDNAs from phylogenetically diverse *Nicotiana* spp. were sequenced and analyzed. Newly discovered NtPRP gene was also included to fully describe relationship among stylar AGPs. *Nicotiana tabacum* ancestral species were added to describe the diversification of these genes that occurred post-hybridization [73]. Due to the overlapping components between II and SI, 120 K sequences were also added to our analysis [30].

## Methods

### Plant material and sequence source

The species used for coding sequences of the PELPIII included: *N. kawakamii* (PI# 459106; NCBI sequence accession number: MF278946)*, N. otophora* (PI# 555542; MF278952)*, N. paniculata* (PI# 266380; MF278948)*, N. repanda* (PI# 555551; MF278947)*, N. rustica* (PI# 499174; MF278951)*, N. setchellii* (PI# 555557; MF278950)*, N. tomentosa* (PI# 574525; MF278949)*, N. stocktonii* (PI# 555538; MF278953), TTS: *N. clevelandii* (PI# 555491; MF278937)*, N. debenyi* (PI# 503320; MF278943) *N. kawakamii* (PI# 459106; MF278936)*, N. miersii* (PI# 555537; MF278940; partial cDNA without signal sequence region)*, N. occidentalis* (PI# 555541; MF402942)*, N. rependa* (PI# 555551; MF40941)*, N. setchellii* (PI# 555557; MF278942)*, N. tomentosa* (PI# 574525; MF278938)*, N. velutina* (PI# 244630; MF278945), *N. paniculata* (PI# 266380; MF278939), *N. otophora* (PI# 555542; MF278944), *N. alata* [76] and *N. obtusifolia* (PI# 555543). Plant material sources were previously described [17]. The above species represent distinct phylogenetic clades of *Nicotiana* spp. [8].

Sequences available from the NCBI database for PELPIII are *N. alata* [U45958.1], *N. sylvestris* [XM_009798359.1], *N. tomentosiformis* [XM_009619111.1] and *N. tabacum* [Z14019.1]; for 120 K: *N. alata* [U88587.1], *N. sylvestris* [XM_009798358.1], *N. tomentosiformis* [XM_009619116.1], *N. bonariensis* [AY886518.1], *N. forgetiana* [AY886517.1], *N. langsdorffii* [AY886516.1.], *N. longiflora* [AY886513.1], *N. plumbaginifolia* [AY886512.1] and *N. tabacum* [AY886511.1]; TTS: *N. alata* [X70441.1], *N. sylvestris* [XM_009760521.1], *N. tomentosiformis* [XM_009604038], *N. tabacum* [Z16403.1] and [Z16404.1].

Contig names of genomic sequences [73, 74] that were identified to carry PELPIII, TTS, 120 K and NtPRP gene sequences can be found in Additional file 1: Table S1.

The alignment of multiple EST sequences of TTS mRNA from expression analysis studies (NCBI dbEST) showed that Z16403.1 (TTS-1) has an additional cytosine (C) at position 687 bp from the start codon that creates a frame shift. Deletion of C^687^ restored the open reading frame making Z16403.1 highly similar to the TTS-2 gene [Z16404.1].

### RNA isolation and cDNA sequencing

Styles were collected from mature flowers, from plants grown in a temperature controlled greenhouse (average temperature of 24.4 °C) with a photoperiod of 14 h day (supplemental light from metal halide lamps) [17], soil mix LC8 (Sun Gro® Horticulture), 20 cm nursery pots and stored at −80 °C. Total RNA was extracted from 3 to 5 styles with the RNeasy Plant Mini Kit (Qiagen, Frankfurt, Gremany). The mRNA was eluted with nuclease-free water and stored at −80 °C until used. First-strand cDNA synthesis was performed based on Pinto and Lindblad [63] using the “combined” method except for a primer (CDS) which was for initiating reverse-transcription instead of a gene specific primer. The cDNA was stored at −20 °C until use. A complete list of primers is shown in Additional file 2: Table S2.

All PCR reactions were performed using Q5 High-Fidelity 2X Master Mix (NEB, Frankfurt, Germany) or Phusion High-Fidelity DNA Polymerase (NEB, Frankfurt, Germany) in a Bio-Rad iCycler Thermal Cycler. Each reaction was prepared according to the user manual for the enzyme or master mix being used. The cycling conditions began with 2 min at 98 °C, followed by 35 cycles of 98 °C for 10 s, 65–68 °C for 30 s plus 72 °C for 30 s and a final extension step at 72 °C for 2 min. A PCR with one gene specific primer and one universal primer at either the 3′-end or 5′-end of the cDNA (3′/5′-RACE) was performed to obtain the full coding sequence information of a gene. A second-round of amplification (nested-PCR) was done using another species- and gene-specific primer.

All PCR products, including 5′ and 3′ RACE were separated on 1% agarose gels and stained with ethidium bromide. The cDNA product was purified using Zymoclean Gel DNA Recovery Kit (Zymo Research Corp, Irvine, USA) and sequenced. Molecular cloning was performed with the use of a NEB PCR Cloning Kit (NEB, Frankfurt, Germany), but only on purified cDNAs that did not produce sequences by direct PCR product sequencing. Sequencing was accomplished by the dideoxy chain termination method by the Genomics Center at the University of Minnesota. The final gene sequence was assembled from multiple (at least three independent) overlapping fragments.

### Sequence analysis

The VSL2B predictor (part of DisProt database of protein disorder) was used to detect protein intrinsically disordered regions [60, 72]. For details on disorder prediction please see Additional file 3: Table S3. Secondary protein structure was predicted using Phyre2 (Protein Homology/analogy Recognition Engine V 2.0; [35]) with default settings using the intensive modeling mode. Signal sequence prediction was performed using the SignalP 4.1 Server [62] with the default settings. *O-*glycosylation patterns were detected using ScanProsite [11, 75] with the amino acid motif [ASTV]-P(1,4)-X(0,10)-[ASTV]-P(1,4) with greedy and no overlap options [28]. Sequence alignment, editing, annotation and manipulation were done using Geneious 8.1.3 software [34], BioEdit v7.2.5 [29] and MEGA7.0 software [39]. Sequencing files were edited and quality checked using 4Peaks software (http://nucleobytes.com/4peaks/). INDEL diversity and average INDEL length was calculated by DnaSP 5.10 software [47]. Gene names for *N. tabacum* include their ancestral donor with S and T indicating similarity to *N. sylvestris* or *N. tomentosiformis,* respectively*.* Evolutionary analysis was performed using MEGA 7.0 software and MUSCLE algorithm (default settings) with minor manual adjustments. The evolutionary history was inferred using the Neighbor-Joining method [67] and the bootstrap test was performed for each tree (500 replicates; [21]). The trees are drawn to scale, with branch lengths in the same units as those of the evolutionary distances used to infer the phylogenetic tree. The evolutionary distances were computed using the Tamura-Nei method [79] and are in the units of the number of base substitutions per site. The rate of variation among sites was modeled with a gamma distribution (shape parameter = 2). The analysis used 12 nucleotide sequences of PELPIII and TTS, and 10 nucleotide sequences of 120 K. Codon positions included were 1st + 2nd + 3rd + noncoding. To calculate the d_N_/d_S_ ratio for PELPIII, TTS and 120 K genes Nei-Gojobori method was used. In both methods, all positions containing gaps and missing data were eliminated. Trees were drawn to scale, with branch lengths in the same units as those of the evolutionary distances used to infer the phylogenetic trees.

## Results

### Identification of a novel NtPRP gene similar to TTS

*Nicotiana tabacum* is an allotetraploid (*2n* = 4*×* = 48) hybrid of ancestral *N. sylvestris* (2*n* = 24; maternal donor) and *N. tomentosiformis* (2*n* = 24; paternal donor). The hybridization took place about 200,000 years ago [45]*.* Genomic sequence data from three inbred genotypes of *N. tabacum* K326 (Flue-cured), TN90 (Burley) and Basma Xanthi (BX, Oriental; [73]) and *N. sylvestris* and *N. tomentosiformis* as species considered to be ancestral donors of *N. tabacum* [74] were searched for sequences similar to the known stylar AGPs. A fourth, closely related gene sequence with significant similarity to TTS but lesser similarity to PELPIII*-*S, PELPIII-T and 120 K-T was discovered and named as NtPRP (Table 1). The NtPRP gene is present in two copies in the *N. tabacum* genome representing the *N. tomentosiformis* (-T) or the *N. sylvestris* (-S) ancestral genomes. NtPRP-T and NtPRP-S are highly similar to each other (94% nucleotide identity; 93% amino acid identity). The NtPRP has a proline-rich N-terminal domain and a C-terminal domain with six conserved cysteine residues that are found in other stylar AGPs. The function of NtPRP gene is not known. A search of the NCBI EST database shows transcript accumulation in seeds (during germination), leaf, flower, two-cell pre-embryo (A comprehensive survey of the *N. tabacum* transcriptome, 2005, NCBI dbEST) and root (*N. tabacum* ‘Hicks Broadleaf’ ESTs, 2007, NCBI dbEST). However, at this point there is no quantitative expression or localization data available for NtPRP.

**Table 1:** Percent identity among NtPRP*,* PELPIII, 120 K and TTS in TN90 variety of *N. tabacum*

	NtPRP-S	NtPRP-T	TTS-S	TTS-T	120 K-T	PELPIII-S	PELPIII-T
NtPRP-S	100 (100)	94 (93)	59 (50)	58 (50)	38 (34)	35 (32)	34 (31)
NtPRP-T	94 (93)	100 (100)	58 (50)	58 (51)	39 (35)	35 (33)	34 (32)

A multiple alignment was performed using Geneious 8.1.3 software [34] using default settings. Numbers are nucleotide and amino acid (in parenthesis) identity (% of bases/residues which are identical)

### Stylar AGPs and NtPRP have similar intron-exon configuration

To identify the ancestral origin of each stylar AGP gene and NtPRP, genomic sequences of both *N. tabacum* ancestral species were analyzed [74]. As expected, two copies of the PELPIII, TTS and NtPRP genes were identified in each of the three *N. tabacum* genomes. A single copy of the 120 K gene was found, which was most similar to the ancestral *N. tomentosiformis*. An NCBI EST database search showed a single gene sequence of the 120 K EST, further confirming the existence of only one copy of this gene in the *N. tabacum* genome. These results suggest that the 120 K-S gene was most likely lost post-hybridization. A single copy of the stylar AGPs and NtPRP genes were found in the *N. sylvestris* and *N. tomentosiformis* sequenced genomes.

**Fig. 1:**
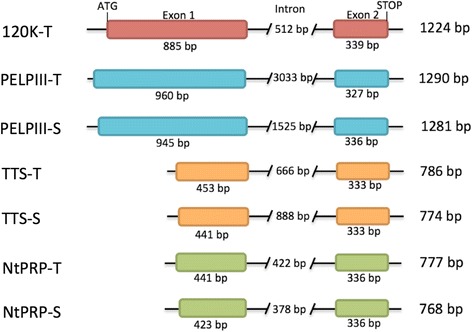
*N. tabacum* intron-exon configuration of the stylar AGP and NtPRP genes. The intron-exon organization of the two ancestral donors, *N. sylvestris* and *N. tomentosiformis,* is nearly identical to *N. tabacum* with minimal INDEL polymorphisms or nucleotide polymorphisms. Sequences were aligned based on second exon sequences, T and S designated *N. tomentosiformis* and *N. sylvestris* ancestral genomes, respectively. Total length of the gene’s coding region is listed on the right. The same intron-exon organization was observed in the newly discovered NtPRP genes (-S and -T)

Each stylar AGP and NtPRP have two exons divided by a single variable-length intron. The exon 1 size ranged from 423 (NtPRP-S) to 960 bp (PELPIII-T), while the single intron ranged from 378 (NtPRP-T) to 3033 bp (PELPIII-T). Exon 2 was conserved in length ranging from 327 (PELPIII) to 339 bp (120 K-T) among all seven genes. Exon 1 in PELPIII and 120 K genes was similar in the length (885 to 960 bp). However, exon 1 in TTS and NtPRP was much shorter (441 to 453 bp; Fig. 1). The PELPIII, 120 K, TTS and NtPRP amino acid sequences near the intron-exon junction (designated based on available genomic sequence data) among all available *Nicotiana* spp. were similar. Amino acid residues encoded near the intron-exon junction are relatively variable in the exon 1 region, but conserved in exon 2 (GAVVKL residues).

### Stylar AGPs and NtPRP have intrinsically disordered and single globular region

Each stylar AGP and NtPRP contains two predicted intrinsically disordered regions (VSL2B predictor; [60]). The IDR1 is at the N-terminal, while IDR2 with a single globular domain is located at the C-terminal. The IDR1 is similar in length among all PELPIII and 120 K proteins, but is significantly shorter in TTS and NtPRP proteins. IDR2 is similar in length among all stylar AGPs and NtPRP.

**Fig. 2:**
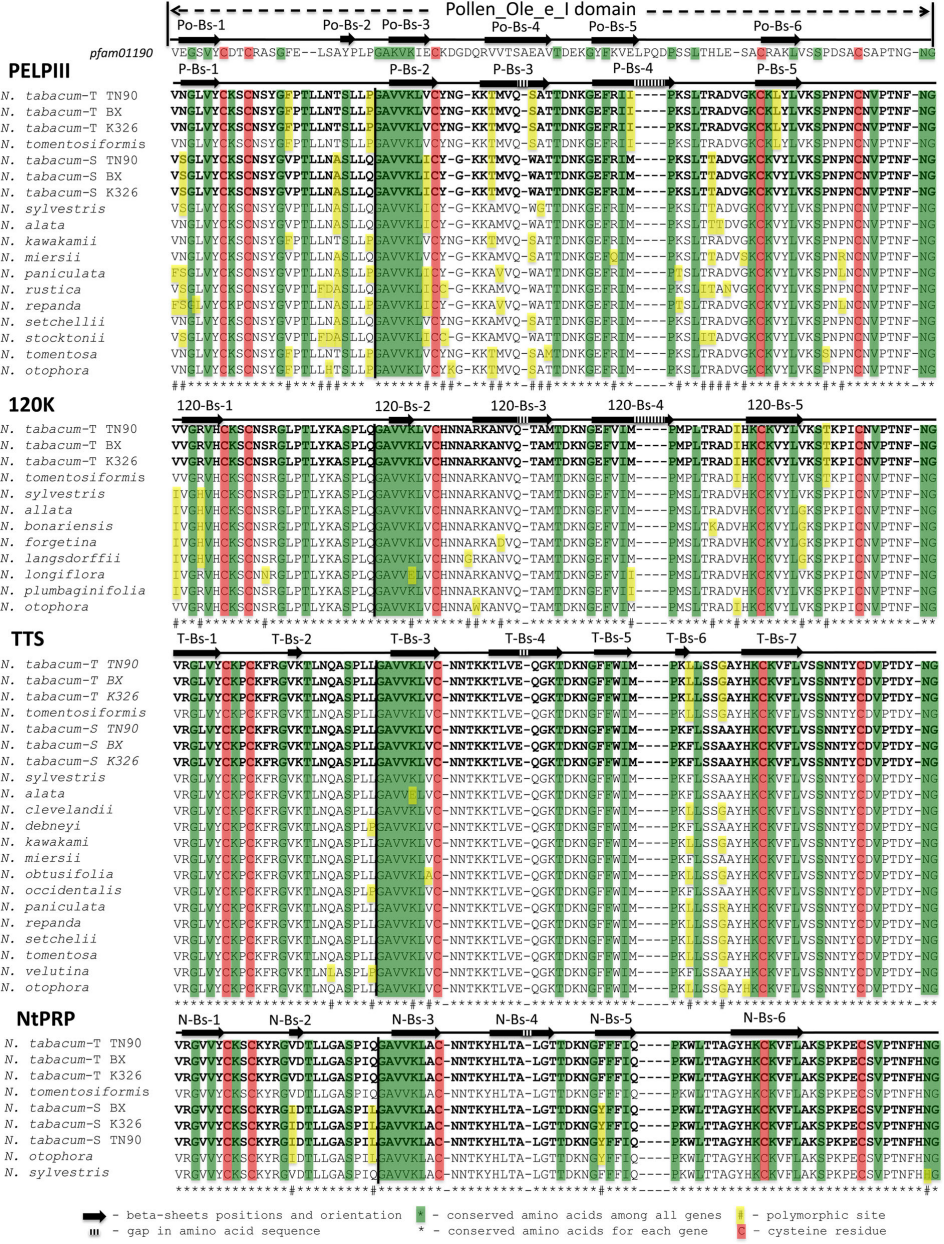
Alignment of the globular region containing an Ole e 1-like domain of stylar AGPs and NtPRP proteins from the *Nicotiana* spp. The predicted secondary structure of the Ole e 1-like domain is indicated above the sequences. *Pfam01190* is a superfamily designation found in the protein families database [24]. The *black vertical line* shows the location of the intron-exon junction that is near the conserved amino acid sequence GAVVKL. Sequences marked in bold are from three *N. tabacum* varieties. Beta sheets (Bs) were designated based on the gene name (PELPIII) and position (1) from left: P-Bs-1

The globular domain of all stylar AGPs and the newly discovered NtPRP have homology to the Ole e 1 superfamily (Fig. 2). The Phyre2 software analysis of the Ole e 1-like domain showed five to seven beta-sheets in the stylar AGPs and NtPRPs. Only short beta-sheet (Po-Bs-2) was characteristic for Ole e 1 domain (*Pfam01190*). Additional two beta-sheets, in TTS and NtPRP short beta-sheets T-Bs-2 and N-Bs-2 are present in close proximity to conserved T-Bs-3 and N-Bs-3, respectively (Fig. 2). Another short beta-sheet (T-Bs-6) is found only in the TTS proteins. This indicates that the amino acid polymorphisms may influence protein folding of the Ole e 1-like domain, due to formation of few, but short, beta-sheets.

### Stylar AGPs and NtPRP are polymorphic among *N. tabacum* genotypes, *N. sylvestris* and *N. tomentosiformis* and *Nicotiana* spp.

Nucleotide, amino acid and INDEL polymorphisms occurred in *N. tabacum* stylar AGPs and NtPRP among three genotypes and *N. sylvestris* and *N. tomentosiformis* (Table 2). Both TTS and NtPRP coding nucleotide sequences were 100% conserved among *N. tabacum* BX, TN90, K326 genotypes. One SNP and one INDEL (27 bp) were found in the BX genotype in PELPIII-S and one INDEL (21 bp) in K326 genotype in PELPIII-T. In 120 K-T one SNP and one INDEL (48 bp) were found in TN90 and K326 genotypes, respectively. When the *N. tabacum* stylar AGPs-S and AGPs-T were compared to the *N. sylvestris* and *N. tomentosiformis* an increased nucleotide and amino acid polymorphism was observed. However, *N. tomentosiformis* NtPRP has 10 SNPs (two amino acid changes) when compared to NtPRP-T of *N. tabacum* genotypes*.* The *N. tomentosiformis* 120 K gene has three SNPs (causing one amino acid change) relative to 120 K-T *N. tabacum* genotypes. The highest nucleotide and amino acid polymorphisms was found among PELPIII genes. The *N. sylvestris* PELPIII had 13 SNPs resulting in 10 amino acid changes and one INDEL (12 bp) when compared to *N. tabacum* genotypes. Out of the 10 amino acid changes, five were proline residues. The INDELs were all found in IDR1 of PELPIII and 120 K and involved the number of proline residues: PSPPPPS (K326, PELPIII-T), PSPL (*N. sylvestris*, PELPIII-S), PPPAKQPSP (BX, PELPIII-S), PPLLPPPPSQPPKQPP (K326, 120 K–T; Table 2). The observed length and amino acid polymorphism of PELPIII and 120 K occurs primarily in the proline-rich IDRs, which could indicate differences in their protein-protein interactions. High sequence conservation of TTS and NtPRP in *N. tabacum* and its ancestral donors may indicate diverging biological roles from the PELPIII and 120 K proteins.

**Table 2:** Summary of stylar AGP and NtPRP polymorphisms among *N. sylvestris* and *N. tomentosiformis* and BX, TN90, K326 genotypes

Gene name	BX	TN90	K326	*N. sylvestris*
PELPIII-S	T/A_492_, INDEL_514–540_	100%	100%	G/C_45_ (L/F), T/C_94_, INDEL_190–201_, C/T_434_ (P/L), G/T_516_ (P/S), C/T_695_ (P/L), G/A_733_ (A/T), A/C_790_ (T/P), A/C_842_ (Q/P), G/A_978_, G/A_982_ (A/T), G/C_998_ (G/A), T/G_1189_(V/F)
TTS-S	100%	100%	100%	A/T_639_
120 K-S	n/a	n/a	n/a	n/a
NtPRP-S	100%	100%	100%	100%
				*N. tomentosiformis*
PELPIII-T	100%	100%	INDEL_223–243_	C/T_514_ (P/S)
TTS-T	100%	100%	100%	T/A_708_
120 K-T	100%	C/T_242_ (L/P)	INDEL_295–345_	T/C_816_, T/A_1097_ (I/K)
NtPRP-T	100%	100%	100%	G/A_15_, G/A_161_, G/C_190_ (A/P), T/C_245_, C/A_264_ (N/K), A/G_267_, G/T_288_, G/T_696_, A/T_70_, A/G_732_

Amino acid changes and relative position are shown in parenthesis. No polymorphism between sequences marked as 100%. The 120 K-S gene copy was not found in any of the three *N. tabacum* genotypes (BX, TN90, and K326). Nucleotide positions are based on nucleotide alignment of four sequences

Cysteine residues are conserved among all PELPIII proteins, one additional cysteine is found in *N. otophora* at position 119. Predicted sequence of *N. clevelandii* obtained from genomic DNA aligns with other PELPIII sequences. The *N. clevelandii* genomic sequence of PELPIII diverges significantly from other PELPIII sequences at amino acid 154. cDNA sequencing produced truncated transcript that was shorter when compared to PELPIII of *N. tabacum*. However, insertion of two nucleotides restores an amino acid sequence that is similar to other PELPIII. Alignment of PELPIII sequence from *N. clevelandii* with the additional nucleotides allows extension of the amino acid sequence and restoration of the cysteine residue at a similar position to PELPIII from *N. tabacum* (Additional file 4: Figure S1)*.* In TTS of *N. repanda* KPPTKPPTYSPSKPPAKSP sequence is duplicated, additionally with KPPT sequence found in three places near region of duplication. Similarly to PELPIII, INDELs are present in IDR1 and IDR2 of TTS (Additional file 5: Figure S2). Analogous features can be seen in 120 K ([30]; Fig. 2), where multiple INDELs were described. Signal peptides from stylar AGPs were obtained from multiple *Nicotiana* spp., and are relatively conserved (with minor amino acid polymorphism) among each stylar AGP (Additional files 4 and 5: Figure S1 and S2). However, each signal sequence is characteristic for each stylar AGP.

Multiple INDELs were found in PELPIII among *Nicotiana* spp., mainly in IDR1 region, with a single amino acid INDEL in the globular region and IDR2 (Additional file 4 Figure S1). In PELPIII of *N. setchellii* and *N. tomentosa* the sequence PPPVKAPSPSPAKQP is repeated and the sequence PAKQP was found in three positions in close proximity. A second repeated sequence PSPAKQSPPPP is found twice in *N. otophora,* but only once in other *Nicotiana* spp. Similarly, there was a short amino acid sequence PSPA found in three positions near each other in *N. otophora* and twice in other species.

To better measure INDEL polymorphisms, INDEL diversity π(i) and average INDEL length were calculated for PELPIII, TTS and 120 K (Table 3). The INDEL diversity was found mainly in the IDR1 of stylar AGPs, with highest INDEL diversity in the PELPIII and 120 K genes, and relatively low INDEL diversity in TTS. Overall the average INDEL length was highest in the 120 K and PELPIII genes, with TTS having much shorter INDELs. This suggests that IDR1 and IDR2 regions are overall least conserved among stylar AGPs and may potentially play a role in regulation of pollen tube growth, possibly differentiating between compatible and incompatible pollen tubes.

**Table 3:** INDEL polymorphism of PELPIII, TTS and 120 K among *Nicotiana* spp.

Gene name	Parameters	IDR1	Ole e 1-like	IDR2	Overall
PELPIII	Average INDEL length (bp)	25.8	3	15	21.4
	INDEL diversity π(i)	4.56	0.5	0.9	6
TTS	Average INDEL length (bp)	14.5	0	21	4.6
	INDEL diversity π(i)	2	0	0.2	1.4
120 K (Hancock et al., 2004)	Average INDEL length (bp)	19.7	0	30	26.4
	INDEL diversity π(i)	4.6	0	0.4	5

Average INDEL length (bp) and INDEL diversity π(i) was estimated using DnaSP [47]

### Stylar AGPs and NtPRP have variable predicted *O-*glycosylation patterns

Stylar AGPs are very heterogenous proteins due to the high degree of variable post- translational modifications, in particular *O*-glycosylation. The *O*-glycosylation is thought to be important in their role as regulators of pollen tube growth [2, 30, 87]. The amino acid motif [ASTV]-P(1,4)-X(0,10)-[ASTV]-P(1,4) was used to predict *O*-glycosylation sites [28]. *O*-glycosylation predictions showed variation in IDRs, with IDR2 being much more uniform among the AGPs among *Nicotiana* spp. No *O-*glycosylation sites were found in the Ole e 1-like domain in any of the AGPs. The IDR2 region of the TTS proteins were unique, lacking predicted *O-*glycosylation sites. When comparing predicted *O-*glycosylation patterns among the same species it is apparent that TTS has a more conserved pattern of predicted *O-*glycosylation sites than the other AGPs. The predicted *O-*glycosylation pattern of 120 K resembles that of the PELPIII. The predicted *O-*glycosylation of NtPRP was conserved and most similar to the TTS gene, however this conclusion is made based on limited sequence data for NtPRP. Relative conservation of TTS and NtPRP *O*-glycosylation patterns among *Nicotiana* spp., when compared to PELPIII and 120 K may relate to the known function of TTS as a regulator of pollen tube growth in general.

### Stylar AGPs and NtPRP are under negative selection

The reproductive AGPs act during pollen tube growth through the style and can participate in prezygotic barriers that maintain species [3, 17, 65, 76]. Multiple methods exist to estimate signatures of selection that could indicate a possible role of the reproductive AGPs in species diversification. The Nei and Gojobori, 1986 algorithm was used to estimate the signatures of selection in stylar AGPs. The d_n_/d_s_ ratio analysis provided evidence that stylar AGPs are under negative selection with d_n_/d_s_ ratios lower than 1. PELPIII and TTS had distinct d_n_/d_s_ ratios on each branch using both a branch and branch-site analysis. Overall d_n_/d_s_ ratio for PELPIII (0.59) is higher than that for TTS (0.29) indicating that negative selection acts differently on TTS within the same set of *Nicotiana* spp. The lowest d_n_/d_s_ value for PELPIII was *N. alata* (0.42) and for TTS was *N. paniculata* (0.09). The highest d_n_/d_s_ values for PELPIII were *N. tabacum*-T (0.89) and *N. tomentosiformis* (0.89) and for TTS was *N. tomentosiformis* (0.81). [30] selected multiple 120 K genes from SI and SC *Nicotiana* spp., based on this selection, d_N_/d_S_ ratio was calculated using Nei and Gojobori algorithm. The overall d_N_/d_S_ ratio was 0.38, which indicates that negative selection also took place for 120 K gene (Additional file 6: Figure S3). In summary, AGP gene d_n_/d_s_ ratio analysis indicate that there has been no positive selection acting on PELPIII, TTS and 120 K genes.

## Discussion

### NtPRP and stylar AGPs intron-exon configuration

Stylar AGPs have been studied because of their role in regulating pollen tube growth [2, 5, 7, 14, 48]. The PELPIII is a specific inhibitor of *N. obtusifolia* and *N. repanda* pollen tube growth [17], the TTS protein promotes pollen tube growth in vivo and in vitro [6] and the 120 K protein is required for *N. alata S*-specific pollen rejection [30]. Despite the progress in functional analysis of stylar AGPs, little is known about the mechanisms of AGP regulation of pollen tube growth and the relationship among stylar AGPs. Availability of genomic sequence [74] of *N. tabacum* allowed discovery of a fourth AGP (named NtPRP) that is similar to the stylar AGPs, and contributes to further understanding of pollen tube-style interactions. The *N. tabacum* PELPIII, 120 K, TTS and the newly discovered NtPRP genes share a very similar intron-exon configuration with two exons, separated by a variable length intron (Fig. 1). Exon 1 protein sequence lacks conservation and may be important in discriminating the distinct functions of the AGPs with specific pollen tube genotypes. Exon 2 protein sequence, which contains the Ole e 1 like domain, is highly conserved among stylar AGPs and NtPRP and may therefore play an important role in pollen – style biology. The NtPRP may have a similar function to TTS serving as redundancy in promoting pollen tube growth or it may have additional functions given its mRNA accumulation can occur outside the mature style. The mRNA accumulation of NtPRP suggests a potential role of this gene in leaf, seedling and root tissues. Identification of NtPRP provides a new opportunity to investigate its role in pollen tube growth regulation.

### Stylar AGPs as interactors

**Fig. 3:**
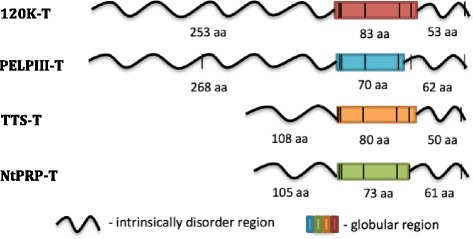
Protein structure of the stylar AGPs and NtPRP in *N. tabacum*. Only IDRs with >50% of disorder disposition are shown. Predictions using other *Nicotiana* spp. stylar AGPs and the S genes of *N. tabacum* had identical structure and are not shown. Regions of predicted intrinsic disorder disposition are shown as a wavy line

Secondary structure analysis showed that stylar AGPs and NtPRP have a single globular region that contains the Ole e 1 - like domain (Fig. 2) and two intrinsically disordered regions, IDR1 and IDR2 (Fig. 3). IDR1 is located in the NTD, but the globular region and IDR2 are located in a region previously referred to as the CTD. As intrinsically disordered regions often interact with two or more proteins [32, 81, 82], variation of the IDRs among *N. tabacum* genotypes may result in altered interactions with pollen tubes, specifically in the rate of pollen tube growth. Similarly, the variation of the IDR among species could result in interspecific incompatible pollen tube growth vs. compatible growth due to a mismatch of AGP and pollen tube protein interaction.

**Fig. 4:**
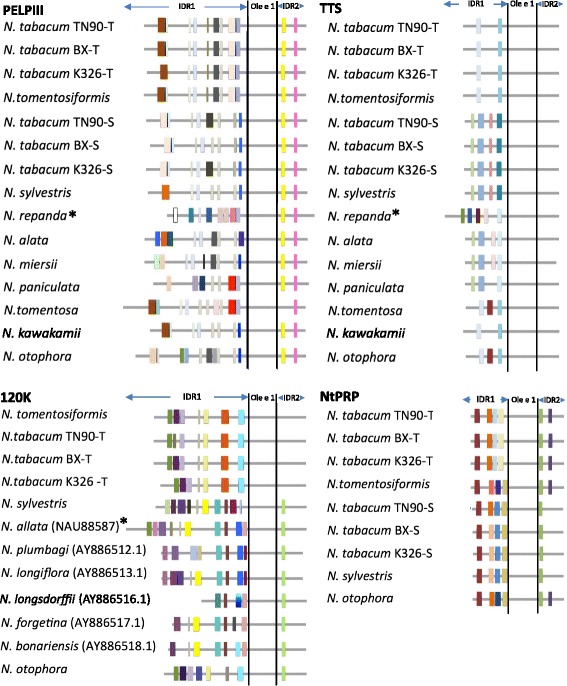
Predicted *O*-glycosylation patterns of stylar AGPs and NtPRP proteins among *Nicotiana tabacum* genotypes and *Nicotiana* spp. Proteins are aligned to the approximate position of the *Ole e 1* domain marked by *black vertical lines*. The species were ordered based on the most parsimonious tree [8] with the exception of *N. tomentosiformis,* which was placed adjacent to *N. tabacum* to allow better comparison of this species to its ancestral version of this gene in *N. tabacum.* The same color represents the same glycosylation site. A different color shade shows amino acid polymorphism found among species within the same *O-*glycosylation site. Colors match the same site only within each gene, not among genes. * - designated species with most distinct *O-*glycosylation pattern among *Nicotiana* spp.

Glycosylation is a significant post-translational modification of AGPs and has a role during protein-protein interactions [40, 41, 58, 59, 80]. The *O*-glycosylation pattern may differ depending on developmental stage or tissue type adding to the complexity of possible protein–protein interactions [13]. An interaction may occur when binding groups of a protein undergo a structural change that facilitates the interaction. *O-*glycosylation predictions indicate that stylar AGPs and NtPRP have variable patterns of *O*-glycosylation among species and slight variation among *N. tabacum* genotypes (Fig. 4). The number of predicted *O-*glycosylation sites varied among species within a gene and was associated with the variable length of the IDR1 sequence. Amino acid sequence polymorphisms within predicted *O*-glycosylation sites (at the same relative position) among *Nicotiana* spp. were found, suggesting that *O-*glycosylation sites undergo evolutionary changes and in effect influence recognition of compatible vs. incompatible pollen tubes among *Nicotiana* spp., resulting from different protein-protein interactions.

It is possible that variation of the *O*-glycosylation patterns may be important in the regulation of interspecific incompatibility (PELPIII) or self-incompatibility (120 K). Two species *N. repanda* and *N. alata* showed very distinct *O-*glycosylation patterns of PELPIII. PELPIII inhibits pollen tube growth of *N. repanda* and *N. obtusifolia* when grown in *N. tabacum* styles [17], indicating PELPIII has a role in II. When compared among species, the predicted *O-*glycosylation patterns for *N. repanda* PELPIII and TTS and *N. alata* 120 K have the most distinct patterns of *O-*glycosylation across protein, and most of amino acid polymorphism within each glycosylation site (Fig. 4).

The *O*-glycosylation sites of PELPIII and 120 K among *Nicotiana* spp. have unique patterns and may be integral to the regulation of pollen tube growth determining whether a pollen-pistil interaction is compatible or incompatible thus influencing II (PELPIII) and SI (120 K). In contrast to these results, the *O*-glycosylation pattern of TTS is more conserved among *Nicotiana* spp. The TTS gene from *N. alata*, *N. miersii* and *N. tomentosa*, *N. otophora* share a similar *O*-glycosylation pattern. Additionally, there is conservation of *O*-glycosylation sites among TTS in divergent species. Considering that TTS is known to facilitate pollen tube growth and has no known function in II or SI, the low *O-*glycosylation variation, suggest the important role of TTS in *Nicotiana* spp. NtPRP appears to have a level of conservation similar to that of TTS, when compared among *N. otophora*, *N. sylvestris* and *N. tomentosiformis*, but are unique in PELPIII, 120 K and TTS.

Small *O*-glycosylation differences were found among *N. tabacum* genotypes. When PELPIII amino acid sequence from *N. tabacum* genotypes were compared, there was a difference in K326-T predicted *O-*glycosylation in the first glycosylation site, when compared to the other *N. tabacum* genotypes originating from the same ancestral donor. Lack of the fifth *O*-glycosylation site was recognized in BX-S PELPIII, when compared to the TN90 and K326. There is a difference in amino acid sequence polymorphism in case of the first glycosylated site in *N. sylvestris,* when compared to *N. tabacum* PELPIII-S proteins. TN90-S and K326-S have small changes in amino acid sequence within predicted *O-*glycosylation sites, in addition to a deletion in the K326-S that causes a glycosylation site shift in relation to other *N. tabacum* genotypes. Similarly, INDELs in K326-S protein create difference in the distance of *O-*glycosylation sites, but not in number of glycosylation sites. Those differences were observed among *Nicotiana* genotypes that were changed among genotypes due to selection during breeding, that could be consider genetically highly conserved.

Another feature that can be important for protein-protein interactions is the INDEL diversity found in both IDRs of stylar AGPs and possibly NtPRP among *Nicotiana* spp. (Table 3). The longest and most diverse INDELs are present in 120 K and PELPIII in the IDR1 region among *Nicotiana* spp. TTS has fewer and shorter INDELs overall and the lowest INDEL diversity when compared to PELPIII and 120 K. The presence of INDELS as long as ~26 aa (PELPIII; on average), changes the distance between *O-*glycosylation positions in the IDR regions, and may affect the interaction with other proteins.

The Ole e 1-like domain is present in all stylar AGPs and NtPRP and is conserved among other AGPs from many taxa. An Ole e I-like domain is found in the stylar AGP homolog LAT52 protein of *Solanum lycopersicum* [83] and was originally discovered in *Olea europaea* pollen (olive; [66]). Preservation of the Ole e I-like domain among stylar AGPs and NtPRP suggests a conserved and important biological function and can be a key region of protein-protein interaction [12].

Hancock et al. [30] found that *N. plumbaginifolia* (SC) and *N. longiflora* (SC) have a 10-amino acid deletion relative to other *Nicotiana* spp. in the IDR2 (part of CTD) of the 120 K protein and concluded that the deletion would not be expected to inhibit protein folding or function. The predicted pattern of 120 K *O*-glycosylation (Fig. 4) suggest that the deletion in *N. plumbaginifolia* and *N. longiflora* would not change the *O*-glycosylation pattern relative to the diversity of other 120 K proteins at IDR2 (Fig. 4). However, the 10 amino acids may affect interactions with other proteins that may occur in this region due to the shift *O-*glycosylation sites.

### Stylar AGPs are under negative selection

**Fig. 5:**
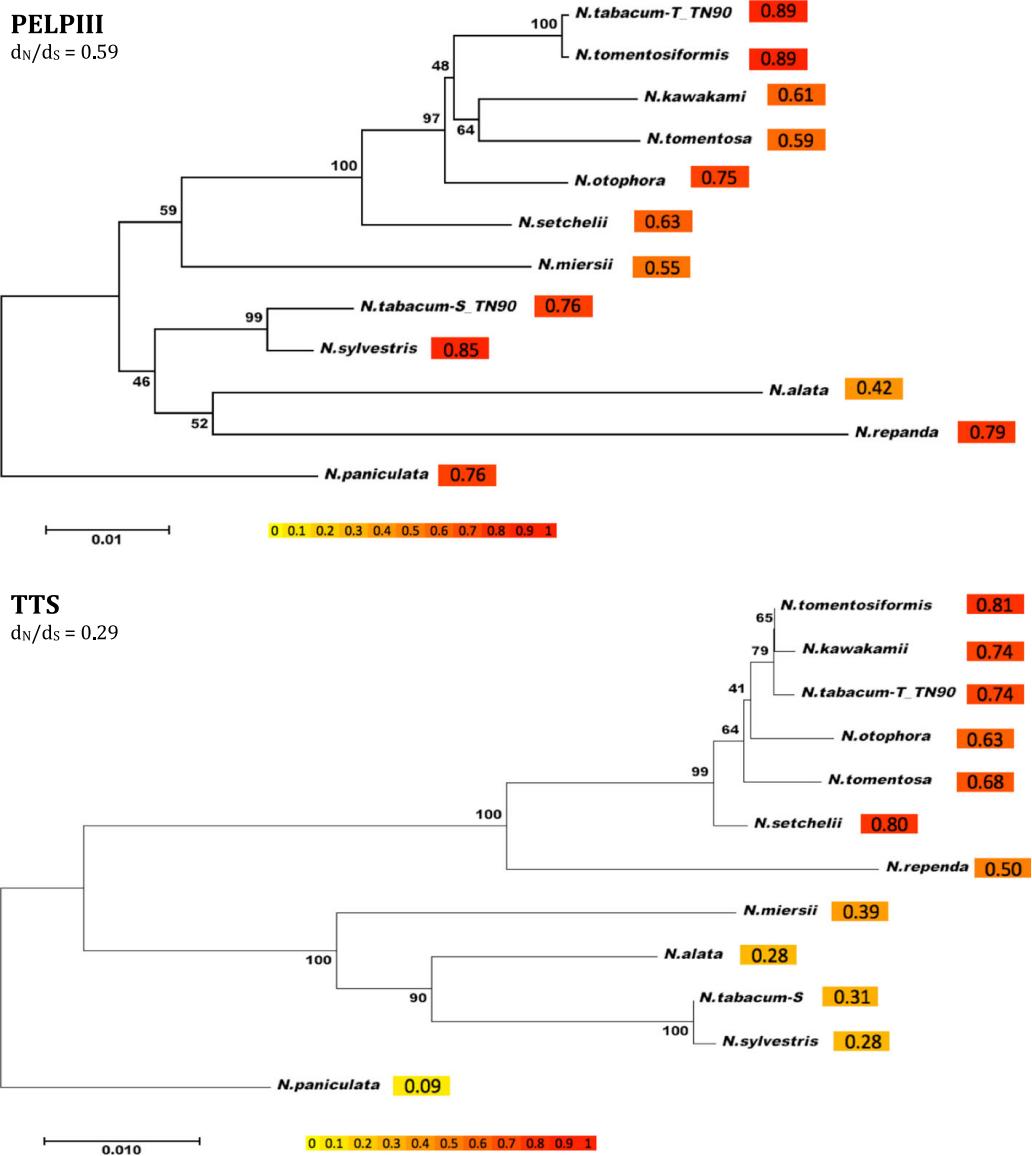
Estimate of the signatures of selection (d_N_/d_S_) in the PELPIII and TTS genes among selected *Nicotiana* spp. The evolutionary distances were computed using the Tamura-Nei method [79] and are in the units of the number of base substitutions per site. d_n_/d_s_ ratios were calculated along each branch using the Nei-Gojobori method. The trees show negative selection (d_n_/d_s_ < 1) in all branches. The color intensity indicates the d_n_/d_s_ ratio. The evolutionary history was inferred using the Neighbor-Joining method [67] and the bootstrap test was performed for each tree (500 replicates; [21]). Bootstrap values are next to each node

PELPIII and 120 K function in II and SI and it is reasonable to assume that the two genes take part in speciation or limit gene flow among *Nicotiana* spp. [17, 19, 30, 51]. The d_N_/d_S_ ratios based on the Nei and Gojbori algorithm provide evidence that the stylar AGPs are under negative selection (Fig. 5). These results are somewhat surprising, considering PELPIII is essential for II and 120 K is essential for SI. However similar results were found with the MID and FUS1 genes that diverged significantly during the evolution of *Chlamydomonas*. The *mid* gene from *C. incerta* carries numerous nonsynonymous and synonymous codon changes compared with the *C. reinhardtii mid* gene, however the estimate of *mid* gene divergence (d_N_/d_S_) using Nei and Gojobori algorithm was relatively low, indicating that there has been no overall positive selection acting on those genes [22]. Our analysis showed that stylar AGPs have different d_N_/d_S_ ratios among species and that each gene undergoes changes differently (Fig. 5).

### Stylar AGPs as paralogs

**Fig. 6:**
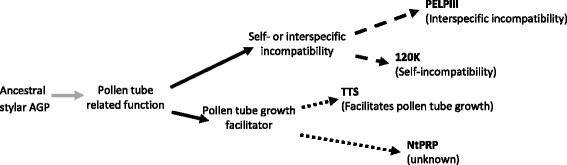
Model of *Nicotiana* spp. stylar AGPs and NtPRP evolution. The *gray arrow* in Fig. 6, indicates an intron introduction into the ancestral AGP and the *black arrows* show a primary duplication resulting in two genes that subsequently diverged from each other. The diversification occurred primarily in exon I, resulting in size and sequence polymorphisms in IDR1 between PELPIII, 120 K, TTS, and NtPRP. The *dashed-line arrows* show a duplication of the self- and interspecific-incompatibility gene that lead to 120 K and PELPIII. The dotted-line arrows show a duplication of the gene involved in facilitating pollen tube growth that lead to TTS and NtPRP. Duplication events resulted in subsequent divergence of the four paralogous AGPs that differentiated further, producing AGPs with diverse reproductive functions in regulation of pollen tube growth (processes of II, SI and SC)

Sequence analysis of stylar AGPs and the newly discovered gene, NtPRP, provided data to propose that the AGP genes have a single common ancestor. It is possible this ancestral gene initially took part in pollen tube growth through style (Fig. 6; Additional file 7: Figure S4 supports this model). After an intron was introduced into this ancestral AGP, it was later duplicated into two genes that diversified to have two separate functions in pollen tube-pistil interactions. One of those duplicated precursor genes was responsible for processes related to self-incompatibility or interspecific-incompatibility (precursor of 120 K and PELPIII genes), and the TTS and NtPRP precursor that likely facilitated pollen tube growth through style. The next two duplication events resulted in the known PELPIII, 120 K, TTS and NtPRP genes.

## Conclusions

Stylar AGPs and the newly discovered NtPRP share similar intron-exon configuration and secondary structure. The location of the single intron, the presence of two intrinsically disordered regions and the conserved Ole e 1-like domain strongly suggest that the stylar AGPs have a common evolutionary origin. These findings create the possibility to manipulate the composition of stylar AGPs and NtPRP as closely related proteins. Additionally, d_n_/d_s_ ratios calculated with use of Nei-Gojobori method for PELPIII, TTS and 120 K provide evidence that these genes are under negative selection. Future studies that swap domains among PELPIII, 120 K, TTS and NtPRP will show how the divergent and conserved domains of the AGPs could influence regulation pollen tube growth and II among *Nicotiana* spp.

## Additional files

Additional file 1: Table S1. Contigs that contained stylar AGPs and NtPRP sequences. (DOCX 14 kb)

Additional file 2: Table S2. Primers used during cDNA synthesis, 5′ and 3′ RACE and gene specific product amplification. (DOCX 20 kb)

Additional file 3: Table S3. A. Summary of the disorder disposition prediction for stylar AGP and NtPRP. (DOCX 53 kb)

Additional file 4: Figure S1. PELPIII amino acid sequence multialignment. (DOCX 2232 kb)

Additional file 5: Figure S2. TTS amino acid sequence multialignment. (DOCX 1658 kb)

Additional file 6: Figure S3. Estimate of the signatures of selection (d_N_/d_S_) of 120 K gene among selected *N. tabacum* species [30]. (DOCX 88 kb)

Additional file 7: Figure S4. Neighbor-Joining tree for stylar AGPs and NtPRP. (DOCX 2863 kb)
