# Supplementary material for: Polymorphism and structure of style–specific arabinogalactan proteins as determinants of pollen tube growth in Nicotiana
Source: BMC Evol Biol. 2017 Aug 10;17:186. doi: 10.1186/s12862-017-1011-2 (PMC5553597; doi:10.1186/s12862-017-1011-2)
Supplement: Supplementary file 1 — Contigs that contained stylar AGPs and NtPRP sequences. (DOCX 14 kb) [file 12862_2017_1011_MOESM1_ESM.docx]

Table S1. Contigs that contained stylar AGPs and NtPRP sequences.

|  | **PELPIII** | **TTS** | **120K** | **NtPRP** |
| --- | --- | --- | --- | --- |
| ***N. tabacum*** **TN90** | AYMY01S058331.1-S AYMY01S030632.1-T | AYMY01S124875.1-S AYMY01S057099.1-T | AYMY01S030632.1-T | AYMY01S025157.1-T AYMY01S107359.1-S |
| ***N. tabacum* K326** | AWOJ01108192.1-S AWOJ01224513.1-T | AWOJ01025088.1-S AWOJ01333785.1-T | AWOJ01101584.1-T | AWOJ01220085.1-S AWOJ01284797.1-T |
| ***N. tabacum* BX** | AWOK01S268742.1-T AWOK01S065924.1-S | AWOK01S242548.1-T AWOK01S148835.1-S | AWOK01S153806.1 | AWOK01S022862.1-T AWOK015550.1+AWOK01S1035314.1-S |
| ***N. sylvestris*** | ASAF01099377.1 | ASAF01132704.1 | ASAF01099377.1 | ASAF01153951.1 |
| ***N. tomentosiformis*** | ASAG01054525.1 | ASAG01139902.1 | ASAG010545257.1 | ASAG01029113.1 |
| ***N. otophora*** | AWOL01S0042857.1 | AWOL01S0026666.1 | AWOL01S0025592.1 | AWOL010S0018102.1 |

*Nicotiana* contigs from Sierro et al., 2013 and Sierro et al., 2014. –S and –T designate sequences that are homologous to the *N. tabacum* ancestral species of *N. sylvestris* (-S) and *N. tomentosiformis* (-T), respectively.
