# Supplementary material for: Polymorphism and structure of style–specific arabinogalactan proteins as determinants of pollen tube growth in Nicotiana
Source: BMC Evol Biol. 2017 Aug 10;17:186. doi: 10.1186/s12862-017-1011-2 (PMC5553597; doi:10.1186/s12862-017-1011-2)
Supplement: Supplementary file 2 — Primers used during cDNA synthesis, 5′ and 3′ RACE and gene specific product amplification. (DOCX 20 kb) [file 12862_2017_1011_MOESM2_ESM.docx]

**Table S2. Primers used during cDNA synthesis, 5’ and 3’ RACE and gene specific product amplification.**

| **Name** | **Sequence (5' → 3')** | **Description** |
| --- | --- | --- |
| **CDS*** | CCAGTGAGCAGAGTGACGTTTTTTTTTTTTTTTTTVN | cDNA synthesis primer (1^st^ strand cDNA) |
| **TSO#** | GTCGCACGGTCCATCGCAGCAGTCACAGGGGG/3SpC3/ | RT reaction template-switch oligonucleotides with a 3' C3-spacer |
| **UP-LA*** | GAGGACTCGAGCTCAAGCCCAGTGAGCAGAGTGACG | Universal primer for first-round PCR in PELP III 3'-RACE |
| **UP-SA*** | GAGGACTCGAGCTCAAGCC | Universal primer for nested PCR in PELP III 3'-RACE |
| **UP-LS#** | ACGCTGACGCTGAGCCTACCTGACGTCGCACGGTCCATCGCAGCAGTC | Universal primer for first-round PCR in 5'-RACE |
| **UP-SS#** | ACGCTGACGCTGAGCCTACCTGAC | Universal primer for nested PCR in 5'-RACE |
| **UP-LA2*#** | ACGCTGACGCTGAGCCTACCTGACCCAGTGAGCAGAGTGACG | Universal primer for first-round PCR in TTS 3'-RACE |
| **UP-SA2*#** | ACGCTGACGCTGAGCCTACC | Universal primer for nested PCR in TTS 3'-RACE |
| **L1-neo** | CTAGTACTTGGCTCATTCTCAAAG | *Nicotiana* PELP III sense primer |
| **N.mie_GSP3** | CGACATTAATACCACTTATTCCTCC | *N. miersii* PELP III sense primer |
| **N.oto_GSP3** | ATGGGCCTACATTCGTGCTAC | *N. setchellii* PELP III sense primer |
| **N.tab_GSP3** | GTCGACAATACCACTTATTCCTCC | *N. paniculata* and *N. rustica* PELP III sense primer |
| **SeqFW-1** | GCCACCAGTTGCTTATCC | *Nicotiana* PELP III sense primer |
| **R1** | AGAAGAGGCGATTTCTTAGC | *Nicotiana* PELP III antisense primer |
| **SeqFW-2** | TGTAAATCCTGCAACAGC | *Nicotiana* PELP III sense primer |
| **SeqFW-3** | ACAGACAACAAAGGTGAGTTTC | *Nicotiana* PELP III sense primer |
| **GSP5** | GTGGTGATGGCTTACATGGTGAAGG | *Nicotiana* PELP III antisense primer |
| **NGSP5A** | AGGCAMATCAGGKAAGTTGGGAATC | *Nicotiana* PELP III antisense primer |
| **NGSP5B** | GCCGGTAAAAGGAGGAATAAGTGG | *Nicotiana* PELP III antisense primer |
| **L2/PIII L2** | ATTCCCAACTTMCCYGATKTGC | *Nicotiana* PELP III sense primer |
| **R2/PIII R2** | GAAACTCACCTTTGTTGTCTGTC | *Nicotiana* PELP III antisense primer |
| **SeqFW-4** | TGTCCCAACAAATTTCAATG | *Nicotiana* PELP III sense primer |
| **TL1** | GGCCCTTGTTCTTTTTCAGC | *Nicotiana* TTS sense primer |
| **TR1** | GCACCTTCTTTGAGGGTTCG | *Nicotiana* TTS antisense primer |
| **T5A** | AACCCCTCTAAACTTGCAMGGYTTGC | *Nicotiana* TTS antisense primer |
| **T5B** | AGRCCACGKACWGCTACAGGTTTCC | *Nicotiana* TTS antisense primer |
| **T3A** | TAGGAAACCTGTAGCWGTMCGTGG | *Nicotiana* TTS sense primer |
| **T3C** | GCAACAACACAAAGAAGACATTAG | *Nicotiana* TTS sense primer |

Methods were based on Pinto and Lindblad (2010). Degenerate base symbols are based on nomenclature for incompletely specified bases in nucleic acid sequences (Cornish-Bowden, 1985). * designate primers by Scotto-Lavino et al., 2006, # designate primers by Pinto and Lindbland, 2010.
