# Supplementary material for: Polymorphism and structure of style–specific arabinogalactan proteins as determinants of pollen tube growth in Nicotiana
Source: BMC Evol Biol. 2017 Aug 10;17:186. doi: 10.1186/s12862-017-1011-2 (PMC5553597; doi:10.1186/s12862-017-1011-2)
Supplement: Supplementary file 3 — A. Summary of the disorder disposition prediction for stylar AGP and NtPRP. (DOCX 53 kb) [file 12862_2017_1011_MOESM3_ESM.docx]

**Table S3. A. Summary of the disorder disposition prediction for stylar AGP and NtPRP.**

The columns are the number of amino acids for each region . Prediction of intrinsically disordered regions was performed using Disprot (Obradovic et al., 2005) with a window size of 20 amino acids to eliminate regions of low confidence to be intrinsically disordered.
