# Supplementary material for: Polymorphism and structure of style–specific arabinogalactan proteins as determinants of pollen tube growth in Nicotiana
Source: BMC Evol Biol. 2017 Aug 10;17:186. doi: 10.1186/s12862-017-1011-2 (PMC5553597; doi:10.1186/s12862-017-1011-2)
Supplement: Supplementary file 4 — PELPIII amino acid sequence multialignment. (DOCX 2232 kb) [file 12862_2017_1011_MOESM4_ESM.docx]

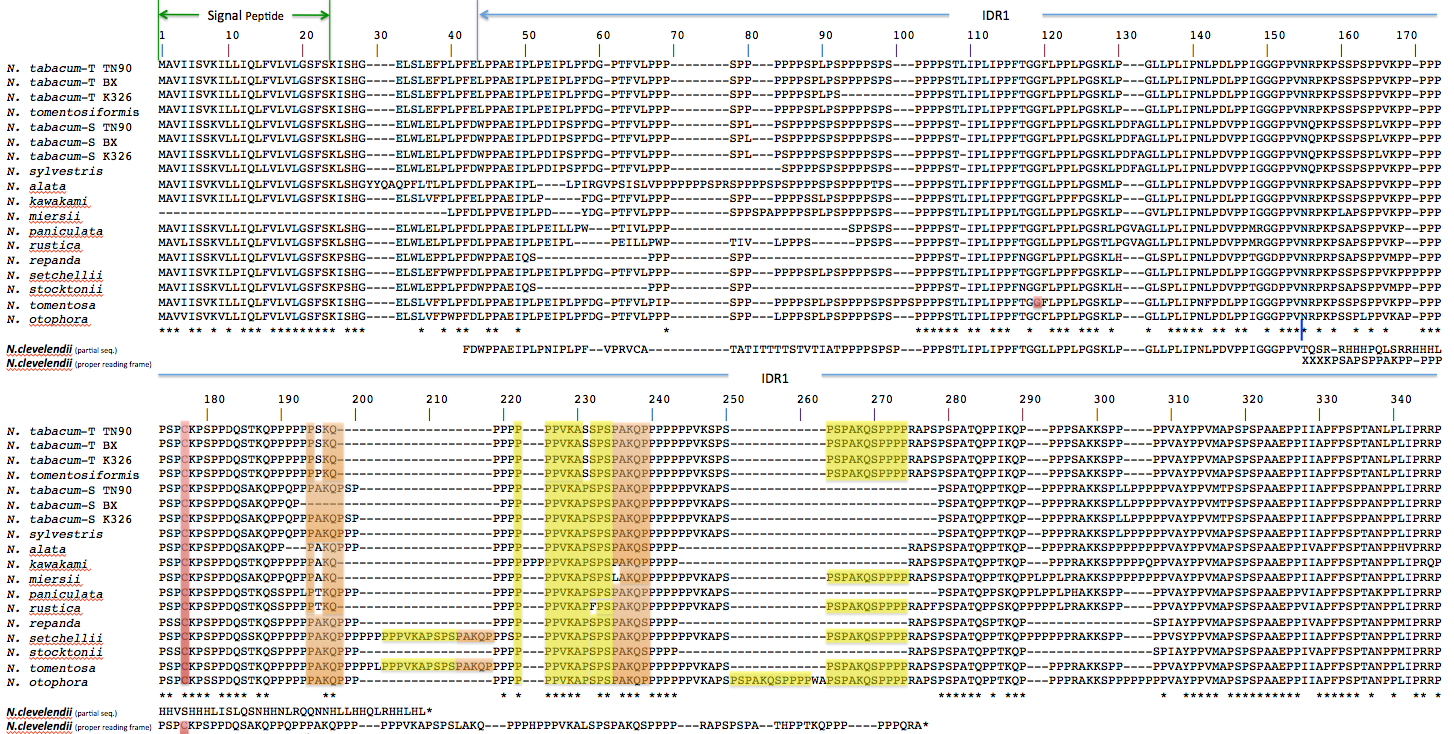

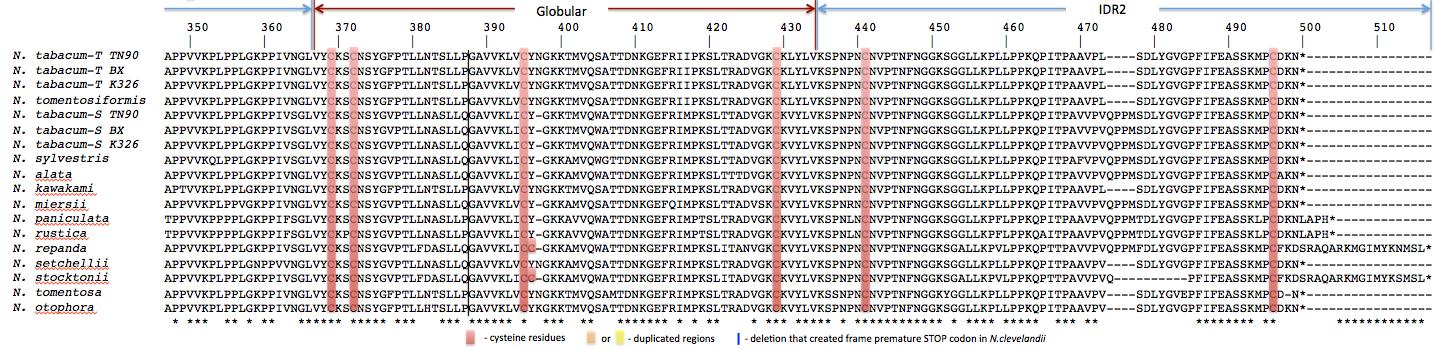

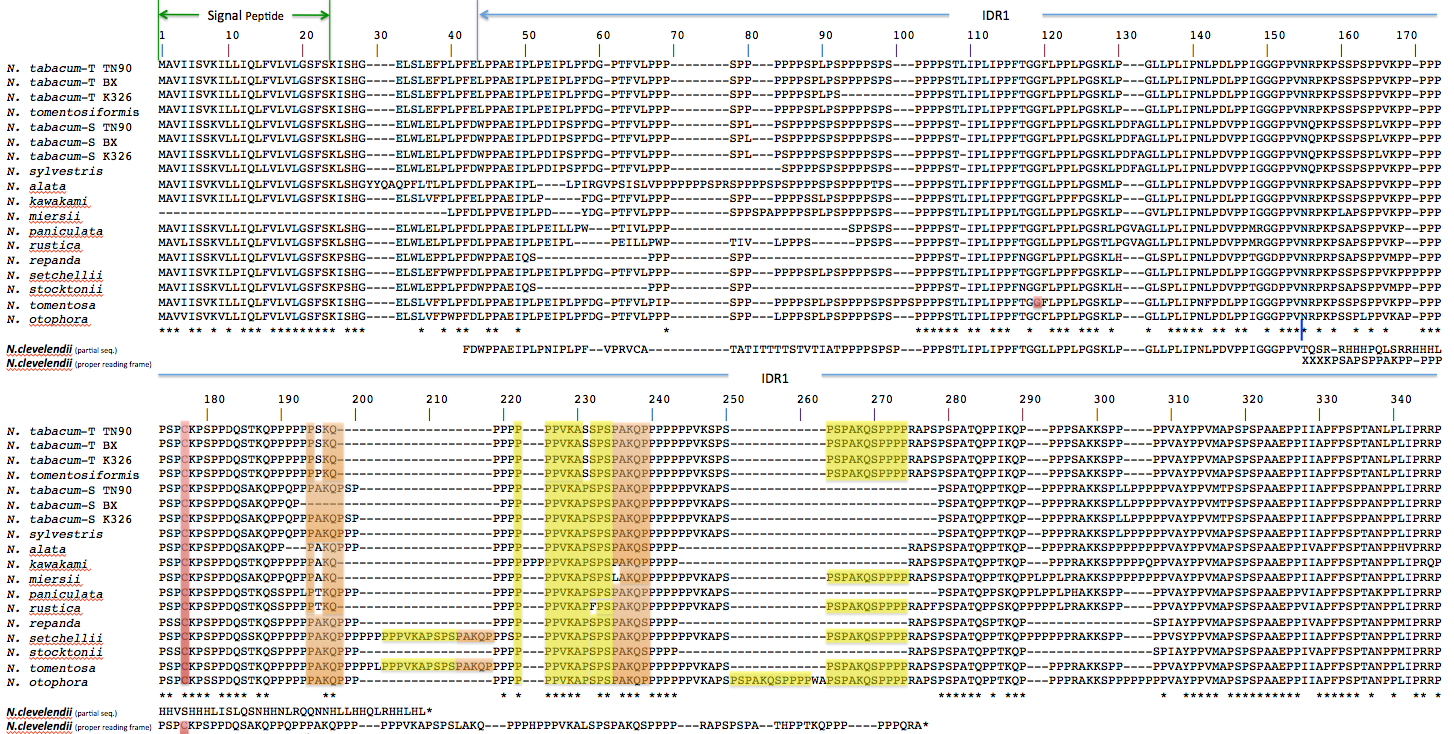

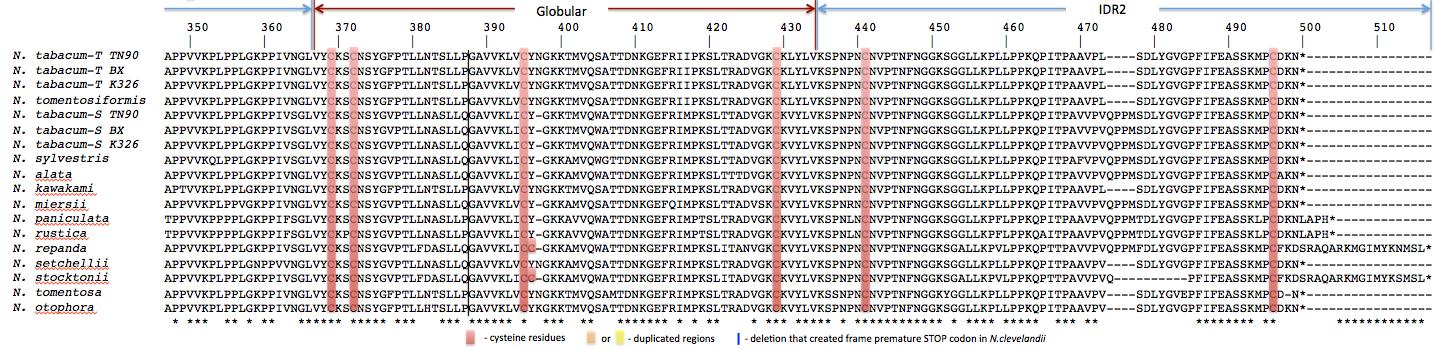


***N. clevelandii*** *(*partial sequence)

***N. clevelandii*** (restored sequence)

***N. clevelandii*** (partial sequence)

***N. clevelandii* (**restored sequence)

* - residues that are 100% conserved among *Nicotiana* spp.

- position of deletion that created frame shift and premature STOP codon in *N. clevelandii*

- cysteine residues

- duplicated regions

or


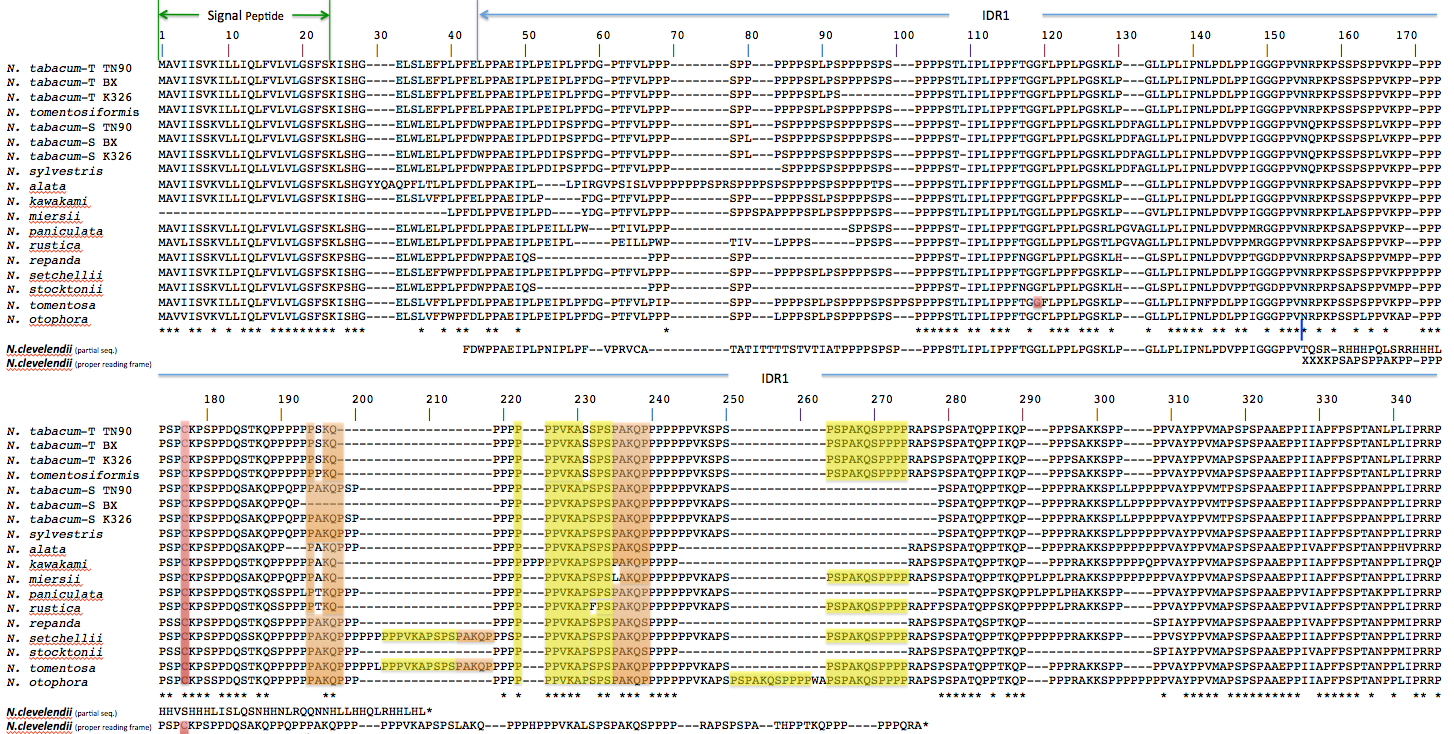


111

**Figure S1. PELPIII amino acid sequence multialignment**. For *N. clevelandii,* both the partial and restored PELPIII sequences are shown; cysteine residue in black border). The black vertical line at residue 387 shows the position of the intron-exon junction. Signal sequence, IDR1, globular and IDR2 regions are indicated above sequence alignment. Sequence alignment was performed using Geneious 8.1.3 software (default settings).
