# Supplementary material for: Polymorphism and structure of style–specific arabinogalactan proteins as determinants of pollen tube growth in Nicotiana
Source: BMC Evol Biol. 2017 Aug 10;17:186. doi: 10.1186/s12862-017-1011-2 (PMC5553597; doi:10.1186/s12862-017-1011-2)
Supplement: Supplementary file 5 — TTS amino acid sequence multialignment. (DOCX 1658 kb) [file 12862_2017_1011_MOESM5_ESM.docx]

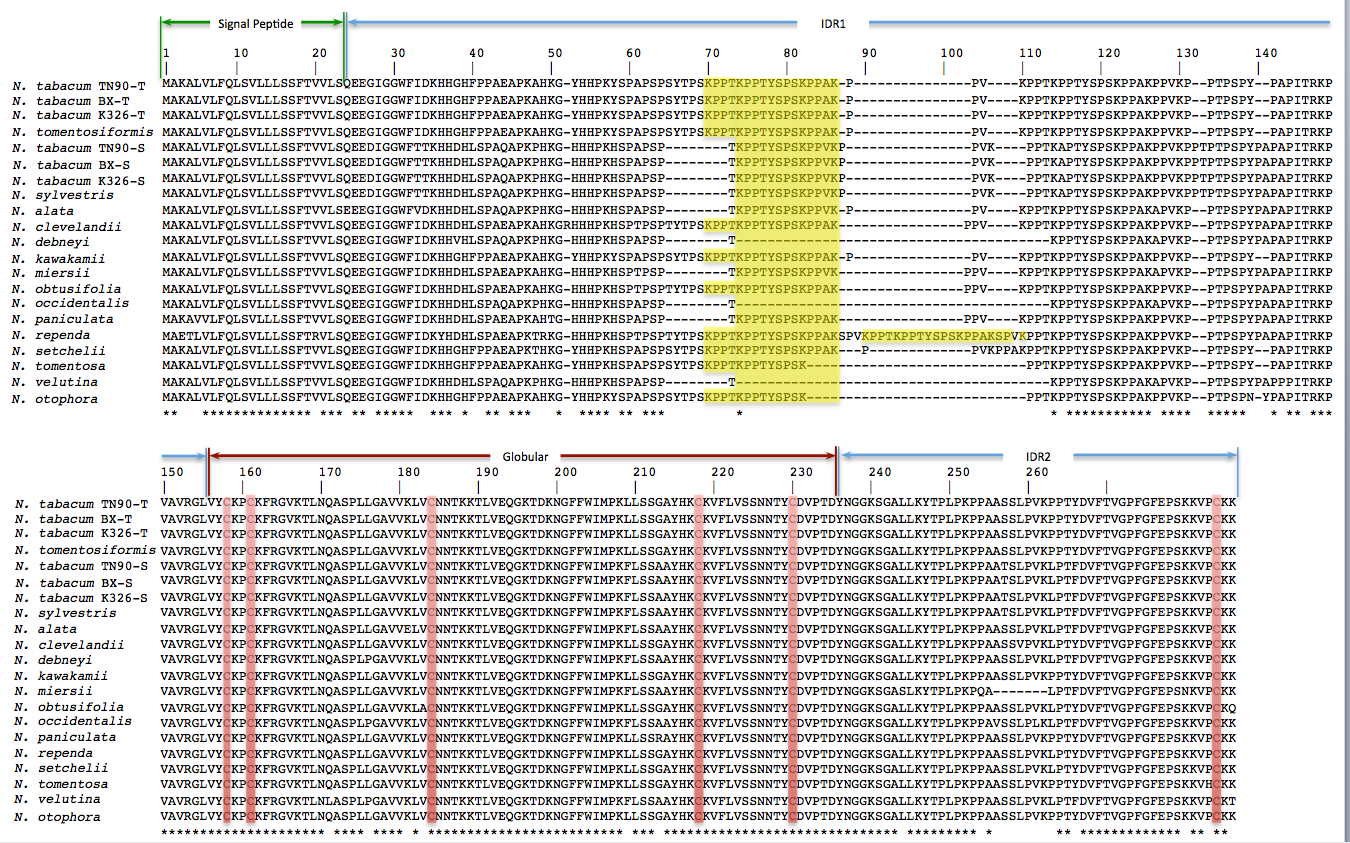

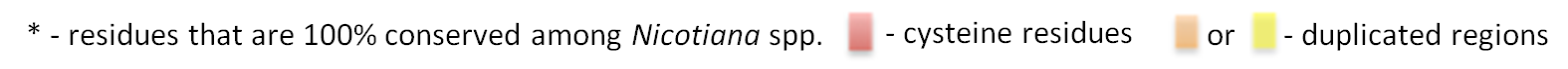


**Figure S2. TTS amino acid sequence multialignment.** Regions of repeated sequences are shaded yellow. In *N. repanda,* KPPTKPPTYSPSKPPAKSP sequence is duplicated, additionally with KPPT sequence found in three places near major INDEL of TTS. Cysteine residues (shaded red) are conserved among all TTS proteins. Black vertical line shows position of intron-exon junction Signal sequence, IDR1, globular and IDR2 regions are indicated. Sequence alignment was performed using Geneious 8.1.3 software (default settings).
