# Supplementary material for: Polymorphism and structure of style–specific arabinogalactan proteins as determinants of pollen tube growth in Nicotiana
Source: BMC Evol Biol. 2017 Aug 10;17:186. doi: 10.1186/s12862-017-1011-2 (PMC5553597; doi:10.1186/s12862-017-1011-2)
Supplement: Supplementary file 6 — Estimate of the signatures of selection (dN/dS) of 120 K gene among selected N. tabacum species [30]. (DOCX 88 kb) [file 12862_2017_1011_MOESM6_ESM.docx]

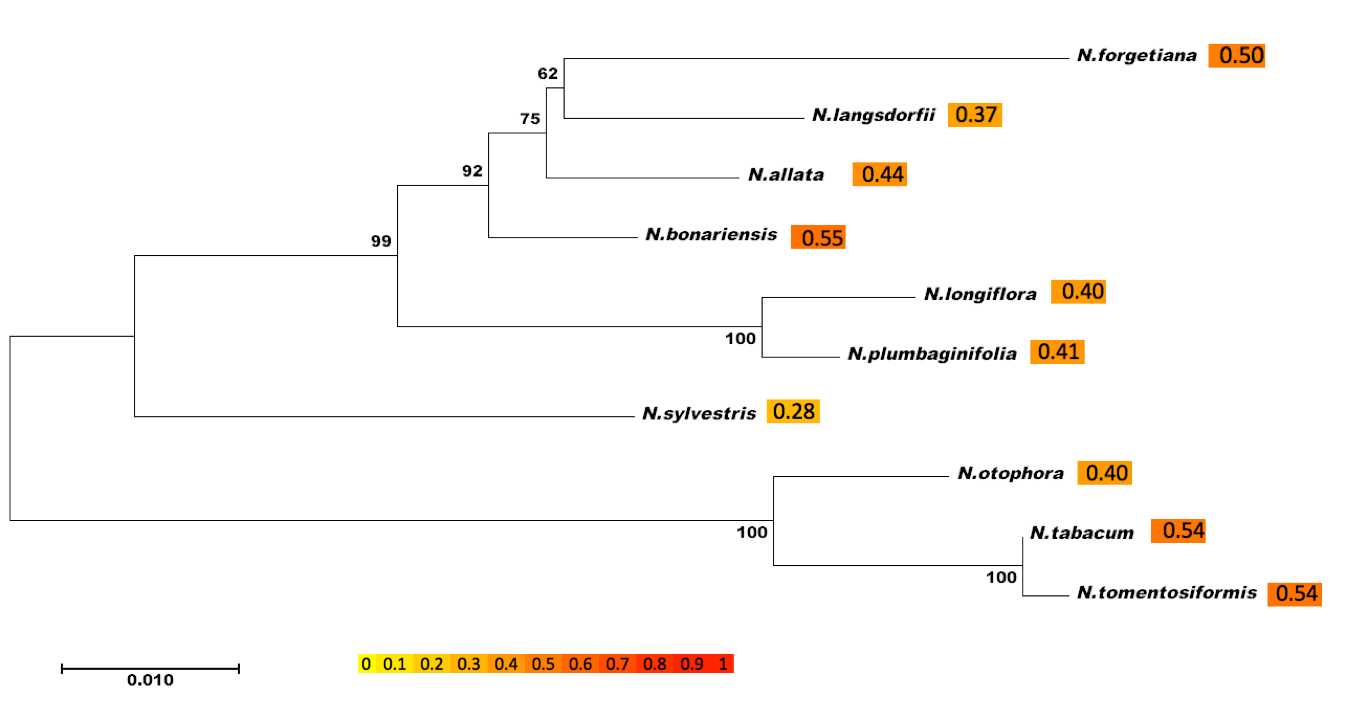
**Figure S3.** **Analysis for positive selection (d_N_/d_S_) of 120K gene among selected *N. tabacum* species (Hancoock et al 2004).** The evolutionary distances were computed using the Tamura-Nei method (Tamura and Nei, 1993) and are in the units of the number of base substitutions per site. d_N_/d_S_ ratios were calculated along each branch using the Nei-Gojobori method. The trees show negative selection (d_N_/d_S_ <1) in all branches and color intensity indicates the d_N_/d_S_ ratio. Bootstrap values are next to each node.

**120K**

d_N_/d_S_ = 0.38
