# Supplementary material for: Polymorphism and structure of style–specific arabinogalactan proteins as determinants of pollen tube growth in Nicotiana
Source: BMC Evol Biol. 2017 Aug 10;17:186. doi: 10.1186/s12862-017-1011-2 (PMC5553597; doi:10.1186/s12862-017-1011-2)
Supplement: Supplementary file 7 — Neighbor-Joining tree for stylar AGPs and NtPRP. (DOCX 2863 kb) [file 12862_2017_1011_MOESM7_ESM.docx]

**
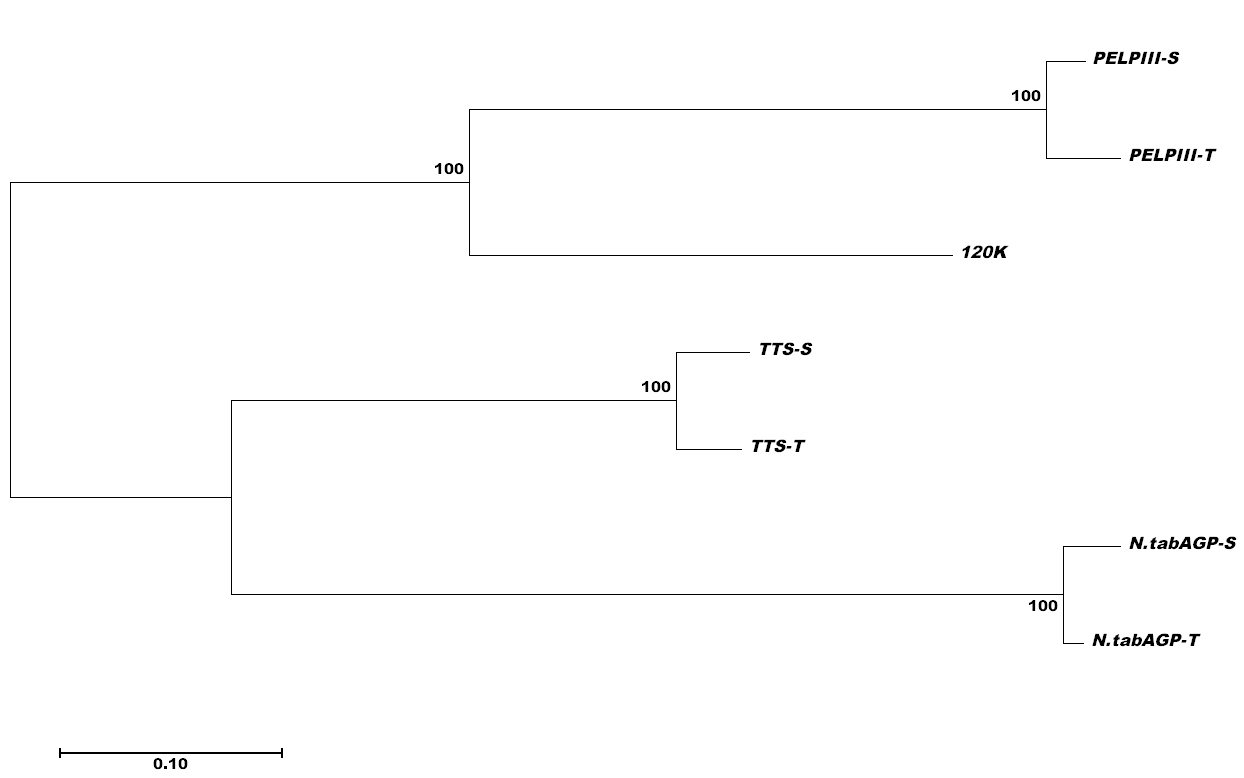
**

**PELPIII-T**

**PELPIII-S**

**120K**

**TTS-S**

**TTS-T**

**NtPRP-S**

**NtPRP-T**

**Figure S4. Neighbor-Joining tree for stylar AGPs and NtPRP.** Phylogenetic tree of the stylar AGPs and NtPRP genes indicate they have a single common ancestor and different rates of evolution. The 120K-S gene was lost after the hybridization event between *N. sylvestris* and *N. tomentosiformis* that produced the allotetraploid *N. tabacum*. Evolutionary analysis was performed using MEGA 7.0 software and the MUSCLE algorithm (default settings) with minor manual adjustments. Trees are drawn to scale, bootstrap values are next to each node.
